# The role of bioactive lipids in attenuating the neuroinflammatory cascade in traumatic brain injury

**DOI:** 10.1002/acn3.51240

**Published:** 2020-12-11

**Authors:** Roy A. Poblete, Marcela Arenas, Nerses Sanossian, William D. Freeman, Stan G. Louie

**Affiliations:** ^1^ Department of Neurology Keck School of Medicine University of Southern California Los Angeles California USA; ^2^ Department of Neurology and Neurosurgery Mayo Clinic Florida 4500 San Pablo Road Jacksonville Florida 32224 USA; ^3^ Department of Clinical Pharmacy School of Pharmacy University of Southern California Los Angeles California USA

## Abstract

Traumatic brain injury (TBI) is a major cause of morbidity, mortality, and economic burden. Despite this, there are no proven medical therapies in the pharmacologic management of TBI. A better understanding of disease pathophysiology might lead to novel approaches. In one area of increasing interest, bioactive lipids known to attenuate inflammation might serve as an important biomarker and mediator of disease after TBI. In this review, we describe the pathophysiology of inflammation following TBI, the actions of endogenous bioactive lipids in attenuating neuroinflammation, and their possible therapeutic role in the management of TBI. In particular, specialized pro‐resolving lipid mediators (SPMs) of inflammation represent endogenous compounds that might serve as important biomarkers of disease and potential therapeutic targets. We aim to discuss the current literature from animal models of TBI and limited human experiences that suggest that bioactive lipids and SPMs are mechanistically important to TBI recovery, and by doing so, aim to highlight the need for further clinical and translational research. Early investigations of dietary and parenteral supplementation of pro‐resolving bioactive lipids have been promising. Given the high morbidity and mortality that occurs with TBI, novel approaches are needed.

## Introduction to Traumatic Brain Injury

Traumatic brain injury (TBI) is a major cause of morbidity and mortality in both the United States (US) and worldwide. From 2006 to 2014, The Centers for Disease Control and Prevention (CDC) estimated that there were 2.9 million US TBI‐related emergency department visits, 288,000 TBI‐related hospitalizations, and almost 57,000 TBI‐related deaths.^1^ Regardless of injury severity, ranging from mild to severe, TBI leads to cognitive, emotional‐behavioral, and physical impairments that result in long‐term functional disability.^2^ In addition to the burden on individuals and families living with TBI, the economic impact is large. The lifetime cumulative medical costs of TBI was recently approximated to be $76.5 billion.^3^, ^4^

To date, there are no proven or FDA‐approved pharmacologic therapies that influence functional outcomes in TBI. Among several medical therapies being investigated, there is increasing interest in the role of bioactive lipids. In particular, endogenous specialized pro‐resolving lipid mediators (SPMs) of inflammation might serve as reliable biomarkers of disease and potential therapeutic targets. In this review, we describe the pathophysiology of inflammation following TBI, the important role of endogenous bioactive lipids in attenuating neuroinflammation, and their possible therapeutic role in the management of TBI. By doing so, we aim to highlight the need for further clinical and translational research. Given the high morbidity, mortality, and economic costs associated with this disease, novel approaches are urgently needed.

## The Complex Role of Inflammation in Traumatic Brain Injury

Both primary and secondary injuries contribute to short and long‐term outcomes in TBI. Primary injury occurs at the time of impact or acceleration‐deceleration and can involve axonal shearing, contusion, intracranial hemorrhage, and tissue and vascular damage.^5^ The second phase of injury begins within seconds and persists for days to weeks. Because secondary injury takes place after the initial TBI, its deleterious effects are hypothetically preventable. The pathophysiology of secondary injury is complex, including delayed hemorrhage and ischemia, excitotoxicity, metabolic dysfunction, blood–brain barrier (BBB) breakdown, and subsequent cerebral edema that can lead to potentially life‐threatening herniation^6^.

### The Inflammatory Response

Neuroinflammation exacerbates TBI pathology and is a major contributor to secondary cell death.^7^ The inflammatory response begins immediately after injury. Several biochemical changes are triggered by damaged cells, including an excess release of the neurotransmitter glutamate leading to excitotoxicity. Activation of glutamate receptors and voltage‐gated calcium (Ca^2+^) channels result in an influx of Ca^2+^, promoting the release of fatty acids from membrane phospholipids, which can be measured in cerebrospinal fluid (CSF) up to one week post‐TBI.^8^ Polyunsaturated fatty acids (PUFAs) generated from membrane lipids can be preferentially metabolized into bioactive lipids known to modulate inflammation. These include arachidonic acid (AA), docosahexaenoic acid (DHA), and eicosapentaenoic acid (EPA). AA is an omega‐6 PUFA and the most important precursor of proinflammatory eicosanoids like prostaglandin E2 (PGE‐2) and leukotriene B4.

Microglia have been established as the primary cell mediator of the central nervous system (CNS) immune response; however, their exact role in balancing pro‐inflammatory states and pro‐resolving ones is still being investigated. When injured, microglia change from a resting and quiescent state to an activated morphology within 72 h^9^, potentially remaining in this morphology for months.^10^ Early on, activated microglia migrate to the lesion site and act to separate injured and healthy tissue.^11^, ^12^ Propagating the inflammatory cascade, they release large amounts of pro‐inflammatory signaling proteins, like tumor necrosis factor‐α (TNFa), interleukin (IL)‐1b and IL‐6. Additionally, macrophages can also elaborate chemokines such as interferon gamma‐induced protein 10, C‐C motif ligand 2/monocyte chemoattractant protein‐1, reactive oxygen species (ROS), and PGE‐2.^13^ This response is further amplified as extracellular matrix (ECM) degradation and increased BBB permeability allow for the infiltration of additional inflammatory and immune cells such as macrophages, neutrophils, monocytes, and lymphocytes.^14^ For a comprehensive review on the role of microglia in neuroinflammation, please see the article by Rodríguez‐Gómez et al^15^. The emerging use of novel imaging techniques and in vitro culture of microglia has advanced our understanding of the central role these glial cells play in the inflammatory response.^15^

### The Role of Neuroinflammation on BBB Integrity after TBI

The BBB is a highly controlled interface between the intravascular space and the brain parenchyma, regulating the movement of water and immune cells in and out of the CNS. Anatomically, it is comprised of vascular endothelium connected by tight junctions, glial cells, and ECM. After TBI, multiple mechanisms that include neuroinflammation result in degradation of BBB integrity. A permeable BBB facilitates secondary brain injury by acting as a low‐resistance pathway for pro‐inflammatory cytokines, macrophages, neutrophils, and water movement into neuronal tissue.^16^ Permeability of the BBB may be the most important mechanism of secondary brain injury after TBI, as it can lead to symptomatic cerebral edema and life‐threatening herniation syndromes.

The timing of increased BBB permeability and cerebral edema has been described in animal models of TBI. In a rat model by Başkaya et al^17^, permeability followed a biphasic pattern and was greatest within hours of injury, and again after 3 days. The delayed phase of post‐TBI BBB permeability has been attributed to ongoing neuroinflammation. Matrix metalloproteinases (MMPs), a group of enzymes produced by activated microglia in pro‐inflammatory states, targets and cleaves protein components of the ECM, resulting in a loss of BBB integrity. Of special interest, MMP‐9 has been implicated as the primary mediator of delayed BBB breakdown in neurologic injury, with levels observed to be highest at phases that coincide with the timing of increased BBB permeability and cerebral edema.^17^, ^18^ Deletion of the MMP‐9 gene in mice reduces lesion volume and improves motor outcomes after experimental TBI,^19^ providing further evidence for its negative impact on BBB integrity.

Several other processes link inflammation and BBB permeability. Systemic inflammation alone without brain injury leads to BBB disruption by direct endothelial damage and modification of tight junctions. In animal models, exposure of brain endothelial cells to immunogenic lipopolysaccharide (LPS) results in membrane abnormalities and apoptosis.^20^, ^21^ Permeability of tight junctions observed in LPS models occurs in response to endothelial cell synthesis of prostanoids and nitric oxide.^22^, ^23^ After TBI, vascular endothelial growth factor‐A (VEGF‐A) also contributes to the reduced integrity of tight junctions. Upregulation of VEGF‐A from neutrophils and astrocytes acts to reduce expression of tight junction protein claudin‐5, leading to BBB leakage.^24^, ^25^, ^26^ The impact of neuroinflammation on other pathologic processes that regulate BBB function, including oxidative stress, upregulation of aquaporin‐4 channels, and activation of other pro‐inflammatory cells continues to be studied in TBI.

### Additional mechanisms of secondary brain injury linked to neuroinflammation

In addition to altering BBB integrity, neuroinflammation promotes secondary brain injury through energy dysfunction, apoptosis, and microthrombosis formation leading to ischemia. Although mechanisms are poorly understood, metabolic crises after TBI may be the largest factor impacting patient outcomes.^27^ This occurs with or without brain ischemia and is characterized by elevations of CNS lactate and pyruvate that are markers of increased non‐oxidative metabolism due to ischemia or mitochondrial dysfunction.^28^ The pathophysiology of mitochondrial dysfunction after TBI is complex and is thought to be secondary to disrupted mitochondrial signaling, increased oxidative stress secondary to mitochondrial Ca^2+^ influx, and disruption of the electron transport chain.^29^, ^30^, ^31^ Altered mitochondria are then involved in the formation of additional ROS that contribute to oxidative stress and TNF‐mediated cellular apoptosis.^32^, ^33^

As previously discussed, microglia are an important link between inflammatory and metabolic responses after neurologic injury. Acting as resident macrophages of the CNS, they have been observed in two phenotypes following TBI: M1 and M2.^34^, ^35^ While the M1 phenotype is associated with the production of high levels of pro‐inflammatory cytokines, the M2 phenotype is involved in the release of pro‐resolving ones. In LPS rodent models, the M1 phenotype is also associated with measurable changes in metabolism characterized by increased glucose consumption and lactate production.^36^, ^37^ Alternatively, forcing oxidative metabolism promotes the M2 phenotype.^36^, ^38^ Early after TBI, the M1 microglia predominates, promoting inflammation and increasing metabolic demand, leading to a state of metabolic crisis under non‐oxidative conditions.

The uncoupling of metabolic supply and demand leads to ischemic injury of vulnerable tissue. Ischemia is also caused by other processes associated with inflammation. Microthrombus formation is an important cause of secondary ischemic injury after TBI, with inflammatory and thrombotic processes closely linked.^39^ Immediately following experimental TBI, aggregation of activated platelets leads to depression of peri‐lesional blood flow.^40^, ^41^ Post‐TBI thrombogenesis may be independent of both injury severity and pattern of injury,^42^, ^43^ making it difficult to predict and diagnose in clinical practice. Inflammation‐mediated activation of the coagulation cascade and reduced protein C activity is also thought to lead to a hypercoagulable state after TBI, promoting both microthrombosis and large territory delayed cerebral ischemia.^44^

## The Appeal of Modulating Neuroinflammation As a Therapeutic Approach in TBI

Given the many important downstream pathologic effects of inflammation, the ability to modulate it as a therapeutic target in TBI remains appealing. Neuroinflammation has been called a “chronic response to an acute injury,”^45^ persisting years after TBI and potentially leading to the development of long‐term motor dysfunction and cognitive disorders such as Alzheimer’s disease (AD).^46^ Large cohort studies have demonstrated an increased risk of dementia when TBI occurs in either early adulthood or when elderly.^47^, ^48^ The hypothesis that attenuation of acute neuroinflammation after TBI can reduce short and long‐term disability by limiting secondary injury and promoting CNS recovery is attractive and is the basis of several therapeutic approaches described in experimental TBI models and clinical research.

Several unique anti‐inflammatory agents have been investigated in both animal models of TBI and in humans, with a discussion of each agent out of the scope of this review. The use of anti‐inflammatory drugs in TBI has been recently and comprehensively reviewed by Bergold.^49^ Many have explored the impact of anti‐inflammatory therapies on BBB permeability, as permeability and cerebral edema are easily quantified in animal models and can be observed using advanced neuroimaging in humans. Hyperosmolar therapy, including mannitol and hypertonic saline (HTS) have long been used to treat clinically significant cerebral edema. Only more recently, it has been learned that HTS has significant anti‐inflammatory properties, reducing neuroinflammation, microglial activation and downregulating apoptosis in brain injury.^50^, ^51^ Among other recognizable anti‐inflammatory drugs frequently used in clinical practice, magnesium sulfate,^52^ corticosteroids,^53^ and statins^54^ have been studied in human clinical trials but have not demonstrated clinical benefit. Currently, there are no anti‐inflammatory therapeutic approaches that are recommended for human TBI as part of standard care.

Other agents targeted at neuroinflammation have yet to be extensively studied in clinical trials. Minocycline has been investigated as a potential neuroprotectant in TBI because of its numerous anti‐inflammatory properties. In animal models, minocycline reduces levels of pro‐inflammatory cytokines, nitric oxide production, microglial activation, and cerebral edema, and is thought to be an inhibitor of MMPs.^55^, ^56^ It is currently being studied in human traumatic spinal cord and brain injury, with larger clinical trials needed in the future.^57^, ^58^ Several antioxidants have been investigated in animal models of TBI,^59^, ^60^, ^61^ including melatonin, which also exhibits broad anti‐inflammatory properties.^62^, ^63^ With the most notable exception of melatonin, most agents that have been used in clinical and experimental studies are not naturally occurring endogenous compounds, raising questions about their pharmacokinetics, pharmacodynamics, and safety in human subjects.

## Endogenous Bioactive Lipids and Specialized Pro‐resolving Lipid Mediators of Inflammation

Lipids are primarily known as essential components of cellular membranes that are utilized as an alternate source of energy; however, a subset of lipids are increasingly recognized as key mediators of cell growth, adhesion, migration, signaling, and death.^64^ Termed bioactive lipids, they are divided by their biochemical functions into four major families: endocannabinoids, lysoglycerophospholipids/sphingolipids, classical eicosanoids, and SPMs.^65^ Together, they play an important role in regulating pro‐inflammatory and anti‐inflammatory states, while SPMs specifically mitigate neurologic injury by acting as a molecular “stop signal” for pathologic neuroinflammation.

The exact triggering events and mechanism of bioactive lipid generation after TBI is largely unknown; however, it may be similar to what is observed in ischemia‐reperfusion (IR) injury. Massive Ca^2+^ influx after IR has been found to trigger phospholipase A_2_ (PLA_2_) activity, resulting in measurable increases in omega‐3 and 6 PUFAs.^66^, ^67^, ^68^ An accumulation of PUFAs has also been demonstrated in both animal models of TBI^69^ and in patients following TBI.^7^ IR may be one pathologic trigger for free FA production; however, additional triggers may exist and warrant further study.

### Proinflammatory lipids

The activation of resident microglia and infiltration of neutrophils through a disrupted BBB is promoted by lipid mediators, typically octadecanoids,^69^ and eicosanoids derived from AAs such as thromboxanes (TXs), PGs, LTs, and hydroxyeicosatetraenoic acids (HETEs).^70^ These bioactive lipids are generated from the hydrolysis of omega‐6 rich membranes in a pro‐inflammatory state that predominates after injury. Eicosanoids modulate the intensity and duration of the inflammatory response by inducing fever, increasing pain, vascular permeability, blood flow, and soft‐tissue edema, as well as promoting delivery of pro‐inflammatory factors.^70^ Cumulatively, this results in the propagation of an ongoing inflammatory cascade.

### Pro‐resolving lipid mediators after neurologic injury

**Figure 1:**
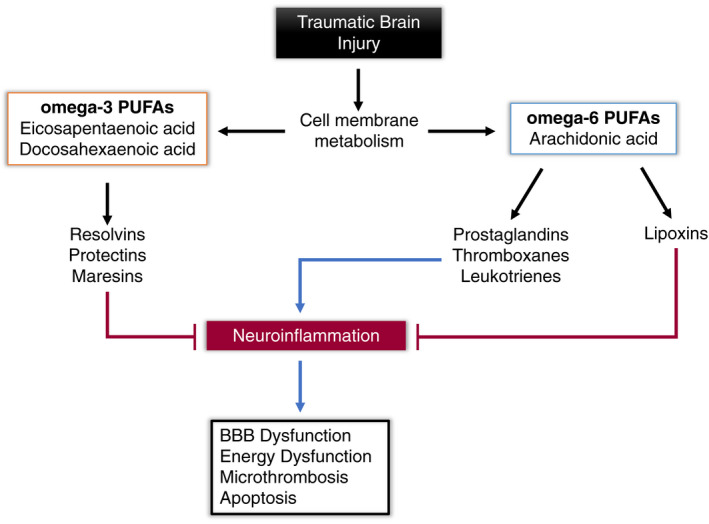
Formation of Bioactive Lipid Mediators and Impact on Neuroinflammation and Downstream Effects. PUFAs, polyunsaturated fatty acids; BBB, blood–brain barrier.

Endogenous SPMs are derived from cellular membrane PUFAs in response to inflammatory states. Notably, those derived from omega‐3 FAs function to resolve inflammation by reducing leukocyte infiltration, promoting killing, and clearance of pathogens, and by stimulating macrophage mediated phagocytosis of cellular debris. They also inhibit the expression of proinflammatory cytokines while inducing production of anti‐inflammatory mediators.^64^ Other SPMs are also simultaneously metabolized from omega‐6 FAs, acting by similar mechanisms to attenuate inflammation and its downstream effects (Figure 1).

The most characterized SPMs include Resolvins (Rv, RvD1, RvE1) and their aspirin‐triggered stereoisomers (AT‐RvD1, AT‐RvE1), Protectins, Maresins, and Lipoxins (LXs). Among these, omega‐6‐derived LXs have been most extensively studied in neurologic disease. During inflammation, lipoxin A4 (LXA4) is formed by two sequential oxygenation reactions of AA catalyzed by lipoxygenases, in particular lipoxygenase‐5 and −12 (LOX‐5 and LOX‐12). LXA4 is an important modulator of granulocyte recruitment into injured tissue. It exerts its transcellular activity through binding and stimulation of G‐protein coupled receptor ALX/FPR2. LXA4 then attenuates inflammation by promoting apoptosis of leukocytes while stimulating efferocytosis—macrophage engulfment of inflammation‐inducing erythrocytes—initiating restoration to homeostasis.^71^ LXA4 has been studied in several chronic disease models that are mediated by inflammation, such as cystic fibrosis and AD. In both conditions, a positive correlation between LXA4 levels and biomarkers of inflammation have been demonstrated.^72^ Administration of SPMs have been shown to ameliorate neural cell death in several animal models of neurologic disease including AD,^73^ epilepsy,^74^ ischemic stroke,^75^ and subarachnoid hemorrhage;^76^ however, their importance in TBI is largely unknown. Recently described, elovanoids represent another class of bioactive lipids that may be important in neuroprotection after brain injury, but more study is needed in TBI.^77^

## Endogenous Lipids as Biomarkers of Disease

As a research tool, lipid biomarkers can determine the extent and intensity of inflammation after TBI and are associated with injury severity. Release of DHA and AAs as a consequence of PLA_2_ activity can be measured at the onset of ischemia and brain trauma.^66^, ^78^ Pilitsis et al^8^ reported that free FA concentrations are significantly higher in CSF 48 hours following TBI as compared to controls without neurological disease. Higher PUFA levels also correlated with TBI severity and poorer clinical outcomes.^8^ In addition to the quantitative measure of AA and DHA following TBI, the ratio of AA:DHA may also be a useful biomarker to understand the overall enzymatic activity that occurs. AA:DHA ratio has been demonstrated to be abnormal in the hippocampi, cortex and plasma of brain‐injured mice.^79^ Human studies have similarly shown significantly reduced ratios of AA:DHA in patients with previous mild TBI. These results suggest preferential metabolism of AA‐containing species over DHA in a chronic inflammatory state. A higher metabolic rate of AA can increase the production of pro‐inflammatory bioactive lipids, acting to sustain the chronic inflammation seen in TBI and TBI‐associated post‐traumatic stress disorder.^80^

Lipid biomarkers of acute TBI in humans remain largely unstudied. While several glial protein biomarkers have been described in TBI, namely S100B, neuron specific enolase and glial fibrillary acidic protein (GFAP) (as reviewed by Dadas^81^), low levels in the acute phase and an inconsistent association with injury severity and outcomes has limited their clinical value. Measurements of other inflammatory cytokines may be too non‐specific to CNS injury.^82^ In contrast, lipids are abundant in the CNS, might produce a unique lipid profile, and due to their ability to cross the BBB, can potentially be rapidly measured in plasma.

Lipidome profiling in animal models of TBI is an emerging field that has the potential to identify important biomarker panels and better understand the pathophysiology of disease. In a recent study using a rat controlled cortical impact (CCI) TBI model, upregulation of PUFAs and PUFA‐containing diacylglycerols was observed, while changes in sphingolipids (SLs) and other membrane phospholipids were also found.^83^ Also using the rat CCI model, Sheth et al^84^ demonstrated that SL measurements were also associated with the severity of TBI inflicted in the rat model. In a second phase of the study, lipidome assays were validated in a human stroke population, suggesting their clinical feasibility to be measured in patients with other types of acute neurologic injury.^84^ Long‐term, altered lipidomes described in both animal models of mild TBI and in corresponding human populations suggest that changes persist even in less severe injury and may have important diagnostic and prognostic implications.^85^, ^86^

## Potential Therapeutic Role of Bioactive Lipids and SPMs in Traumatic Brain Injury

### Evidence from animal models of ischemic and traumatic brain injury

Despite the lack of evidence demonstrating the value of lipid profiles and SPMs as a biomarker in acute TBI, some few and early pre‐clinical research using SPMs as a therapy have been promising. Administration of endogenous bioactive lipids is appealing for several potential reasons. Although the overall biological effect of SPMs is to promote resolution of inflammation, the exact mechanisms of action and downstream effects are likely pleotropic, directly and indirectly targeting the pathophysiology of TBI. Importantly, given the abundance of naturally occurring endogenous lipids and pre‐existing bioactive lipid formulations already in‐market, administration of SPMs is believed to have a positive safety profile over a large dosing range with less drug costs. To date, the effect of administering exogenous SPMs for TBI is largely unstudied and remains an important target for future research. Through multiple mechanisms, administration of bioactive lipids post‐TBI might attenuate acute neuroinflammation and limit chronic derangements associated with long‐term disability.

Considered a low‐risk intervention, early dietary supplementation of anti‐inflammatory PUFAs may reduce the downstream effects of neuroinflammation. Omega‐3 PUFA‐enriched oils can be given in the form of fish oil as a dietary supplement. As a pretreatment and potential neuroprotectant, Wu et al^87^ supplemented the diet of adult rats with 8% fish oil for 4 weeks before fluid percussion TBI and continued treatment for 1 week after injury. Treatment resulted in reduced oxidative stress and improved spatial learning on Morris water maze testing.^87^ Omega‐3 PUFA supplementation implemented before mild TBI can also decrease markers of abnormal cellular energy metabolism.^88^

Dietary supplementation of endogenous pro‐resolving lipids initiated after injury appears to have a beneficial effect on markers of long‐term disease. In an impact acceleration injury model, rats given 30 days of supplementation with either 10 mg/kg/d or 40 mg/kg/d of DHA resulted in significantly increased DHA serum levels that were positively correlated with dose. On immunohistochemical analysis, treatment was associated with a significant decrease in amyloid precursor protein positive axons in a dose‐dependent manner.^89^ In a similar rat model, animals treated with an omega‐3 FA preparation (EPA and DHA, in a ratio 2:1) as a dietary supplementation post‐injury for 30 days decreased levels of caspase‐3, a known mediator of apoptosis.^90^

Dietary supplementation of endogenous lipids might have a beneficial effect on neurotransmission. A microdialysis study demonstrated that 7‐days of fish oil therapy in a rat model of TBI was associated with significant increases in dopamine release into the extracellular space.^91^ These results may partially explain the short‐term neurocognitive and behavioral changes observed after TBI and demonstrate the potential benefit of endogenous lipids.

In human populations of critically ill patients, dietary supplementation can have several practical and pharmacologic limitations. Variability in dosing schedules and impaired gastrointestinal motility and absorption can affect drug bioavailability, while drug–drug interactions and increased metabolic rates can result in lowered drug levels that do not reach therapeutic effect. Parenteral dosing of SPMs has only recently been investigated in animal models of TBI. In a first‐of‐its‐kind study in a brain ischemia‐reperfusion rat model by Wu et al,^92^ intraventricular administration of LXA4 analog LXA_4_ME following middle cerebral artery occlusion resulted in decreased Evans Blue extravasation, reduced expression of MMP‐9, upregulation of tissue inhibitors of metalloproteinase‐1, and reduced final infarct volume. These results suggest that LXA4 can help maintain or restore BBB integrity following acute neurologic injury. The beneficial effect is thought to be partially mediated by attenuation of the pro‐inflammatory cascade. In a mouse model of TBI, Luo et al^93^ demonstrated that a single intraventricular dose of LXA4 downregulates mRNA and protein levels of inflammatory cytokines TNF‐α, IL‐1β, and IL‐6 and reduces BBB breakdown. The authors further demonstrated that this resulted in attenuation of brain edema and a smaller final lesion volume at 7 days after injury.

Alternative SPMs have not been extensively trialed in animal models of TBI. In a recent rat study, RvD1 was administered in the intraperitoneal (IP) space after focal hemicerebellectomy, and then every two days at days 3, 5, and 7. Treatment was found to promote functional recovery and neuroprotection by reducing the activation of Iba‐1 + microglia and GFAP + astrocytes, markers of neurologic injury and a pro‐inflammatory response.^94^ In an additional study, adult mice injured using a midline fluid percussion injury model were administered intraperitoneal RvE1 (100ng daily) or AT‐RvD1 (100ng daily) for 7 consecutive days beginning 3 days prior to TBI. In this diffuse brain injury model, AT‐RvD1 treatment, but not RvE1 was associated with mitigation of motor and cognitive deficits. In contrast, RvE1 treatment, but not AT‐RvD1 demonstrated reduced presence of activated microglia in cortical regions.^95^ Taken together, results from animal models of TBI suggest that the potential benefit of SPM administration is only partially explained by its anti‐inflammatory activity.

### Limited human experiences with bioactive lipid supplementation after traumatic brain injury

Although limited, a few case studies describe the use of lipids in human patients after CNS injury. In one case report, an individual survivor of a mining explosion in the US presented in coma secondary to suspected carbon monoxide toxicity and acute respiratory failure. In addition to fluid resuscitation and hyperbaric oxygen therapy, aggressive dietary supplementation of omega‐3 FA was given during the first 8 days after injury and is believed to have contributed to good neurological outcomes in the patient.^96^ Inspired by this, Lewis et al^97^ administered large amounts of omega‐3 FA (30 mL/day) via percutaneous endoscopic gastrostomy tube to enhance recovery in a teenager with severe TBI after a motor vehicle accident. Despite first impressions of irreversible injury, the patient was ultimately discharged after 4 months and was walking with assistance 2 years later.^97^ More recently, a series of nine cases of patients with severe TBI reported good outcomes using omega‐3 FA supplementation. Shortly after admission, patients were administered twice daily 8.1g of oral omega‐3 oil consisting of a 2:1 ratio of EPA and DHA.^98^ Although these series of cases and anecdotal evidence do not constitute high‐quality evidence, they demonstrate the potential advantages of lipid supplementation, the growing interest in lipids in the treatment of TBI, and the need for larger clinical trials. A summary of investigations of TBI therapy with lipid administration is shown in Table 1.

**Table 1:** Lipids administered in animal models of TBI and in human TBI patients.

Administered Lipid	Subject	Treatment Start	Outcomes
Resolvins^95^	Midline fluid percussion mouse model	Pre‐Injury	AT‐RvD1 treatment, but not RvE1 associated with mitigation of motor and cognitive deficits. RvE1 treatment, but not AT‐RvD1 demonstrated reduced presence of activated microglia in cortical regions
Resolvins^94^	Hemicerebellectomy rat model	Post‐Injury	Reduced activation of Iba‐1 + microglia and GFAP + astrocytes
LXA4^93^	Weight drop mouse model	Post‐Injury	Downregulates mRNA and protein levels of inflammatory cytokines TNF‐α, IL‐1β and IL‐6 and reduces BBB breakdown and attenuation of brain edema
DHA^89^	Impact acceleration injury rat model	Post‐Injury	Significant decrease in APP + axons in a dose‐dependent manner
EPA and DHA^90^	Impact acceleration injury rat model	Post‐Injury	Decreased levels of caspase‐3, a known mediator of apoptosis
Omega‐3 FA^87^	Fluid percussion rat model	Pre‐Injury	Reduced oxidative stress and improved spatial learning on Morris water maze testing
Omega‐3 FA^91^	Controlled cortical impact rat model	Post‐Injury	Significant increases in dopamine release into the extracellular space
Omega‐3 FA^96^	Humans	Post‐Injury	Potential contributor to good neurologic outcome
Omega‐3 FA^97^	Humans	Post‐Injury	Potential contributor to good neurologic outcome
Omega‐3 FA^98^	Humans	Post‐Injury	Potential contributor to good neurologic outcome

LXA4: Lipoxin A4; DHA: docosahexanoic acid; EPA: eicosapentaenoic acid; FA: fatty acid; AT‐RvD1: aspirin‐triggered stereoisomer of Resolvin D1; RvE1: Resolvin E1; GFAP: Glial fibrillary acidic protein; APP: amyloid precursor protein.

In light of limited clinical evidence, many questions remain. Given some of the beneficial effects that early inflammation has on stabilizing and repairing cellular injury, the optimal timing, dosing, and administration route for proposed anti‐inflammatory drugs is unknown. Although administration of lipid compounds is considered relatively safe, human drug trials are needed to establish the therapeutic window of these agents.

#### Future Directions & Conclusions

As post‐TBI inflammation is a key component of secondary brain injury, the understanding of the exact mechanisms of disease will allow us to develop better therapeutic interventions. Additional clinical and translational research is needed to answer several remaining questions about TBI pathophysiology and the importance of endogenous bioactive lipids. In human TBI populations, lipid profiles, specifically SPMs, should be characterized to determine their role as a biomarker of acute disease and their value as a potential therapeutic target in clinical practice.

Early investigations of dietary and parenteral supplementation of pro‐resolving bioactive lipids has been promising; however, further research is needed to describe the specific mechanisms of action for SPMs and optimize administration strategies that will lead to safe and effective clinical trials. TBI is a major cause of death and disability in the US and worldwide. Given the limited treatment options currently available, novel research is urgently needed.

## Conflict of Interest

Dr. Poblete reports grants from NIH, during the conduct of the study; Dr. Louie reports non‐financial support and other from Eyemedix, LLC, outside the submitted work; In addition, Dr. Louie has a patent for lipid‐based compounds licensed to Eyemedix.

## Author Contribution

RP conceived of the thesis of the manuscript. RP and MA conceived the outline and were primary and secondary authors of the manuscript, respectively. Significant intellectual input was received from all authors. NS, WF, and SL were involved in critical manuscript revisions. All authors read and approved the submitted version.
